# Supplementary material for: CryoEM structure of the outer membrane secretin channel pIV from the f1 filamentous bacteriophage
Source: Nat Commun. 2021 Nov 2;12:6316. doi: 10.1038/s41467-021-26610-3 (PMC8563730; doi:10.1038/s41467-021-26610-3)
Supplement: Supplementary file 1 — Supplementary Information [file 41467_2021_26610_MOESM1_ESM.pdf]

CryoEM structure of the outer membrane secretin channel pIV from the f1 filamentous bacteriophage

Rebecca Conners[1,2], Mathew McLaren[1,2], Urszula Łapińska[1,2], Kelly Sanders[1,2], M. Rhia L. Stone[3], Mark A. T. Blaskovich[3], Stefano Pagliara[1,2], Bertram Daum[1,2], Jasna Rakonjac[4] & Vicki A. M. Gold[1,2]*

[1]Living Systems Institute, University of Exeter, Stocker Road, Exeter, UK
[2]College of Life and Environmental Sciences, Geoffrey Pope, University of Exeter, Stocker Road, Exeter, UK
[3]Centre for Superbug Solutions, Institute for Molecular Bioscience, The University of Queensland, Brisbane, Queensland, Australia
[4]School of Fundamental Sciences, Massey University, Palmerston North, New Zealand

*Corresponding author: v.a.m.gold@exeter.ac.uk

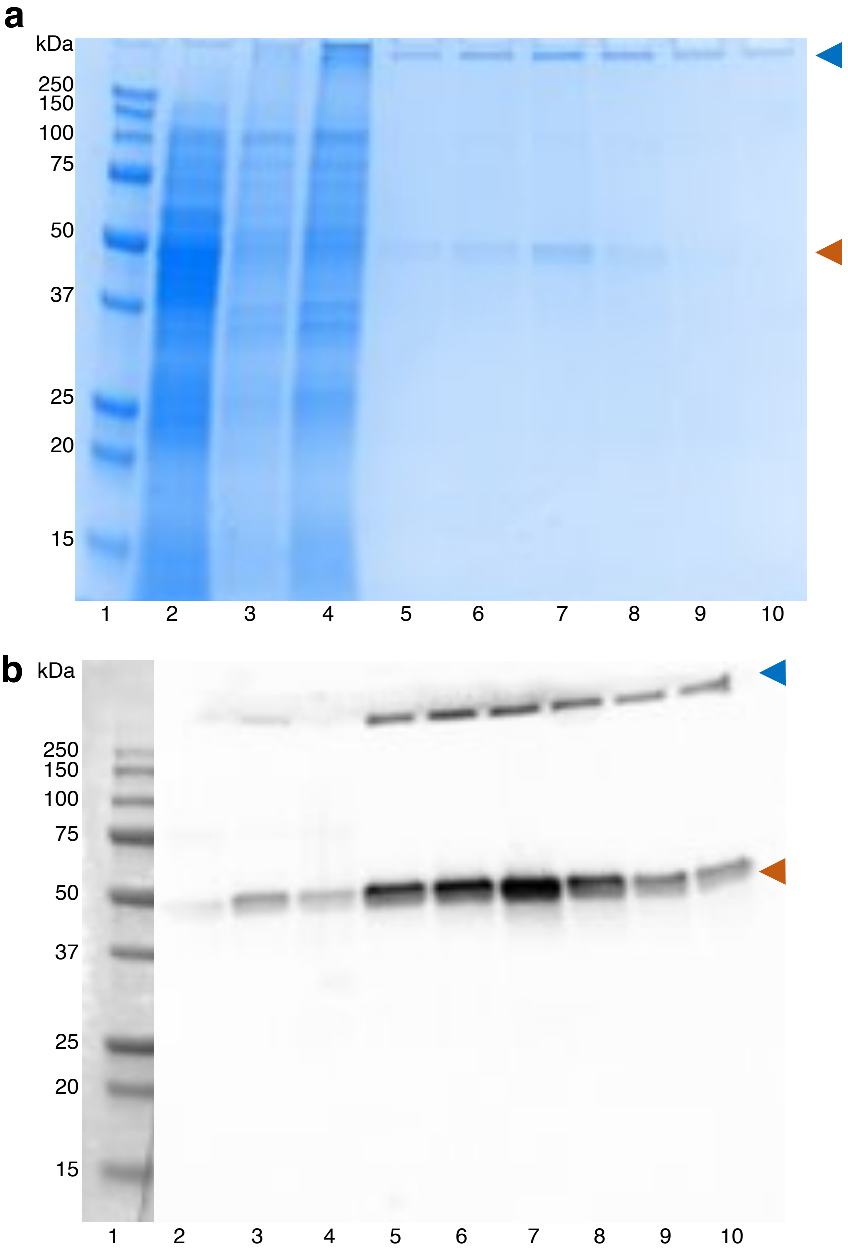

c

Accession	Description	Score	Coverage	# proteins	# unique peptides	# peptides	# PSMs	Area	# AAs	MW (kDa)	Calc. pI
f1pIV	f1pIV sequence	2641.11	77.78	1	40	40	973	3.630E11	405	43.4	5.06
P0A853	Tryptophanase OS=Escherichia coli (strain K12)	248.61	79.83	1	39	39	91	4.152E9	471	52.7	6.23
P0ABB4	ATP synthase subunit beta OS=Escherichia coli (strain K12)	180.28	77.61	1	28	28	56	7.750E8	460	50.3	5.01
P0ABB0	ATP synthase subunit alpha OS=Escherichia coli (strain K12)	120.71	49.32	1	21	22	36	2.840E8	513	55.2	6.13
P0A6U8	Glycogen synthase OS=Escherichia coli (strain K12)	118.33	41.93	1	19	19	38	9.668E8	477	52.8	6.62
P13035	Aerobic glycerol-3-phosphate dehydrogenase OS=Escherichia coli (strain K12)	94.42	56.09	1	23	23	32	1.994E8	501	56.7	7.44
P0A6F3	Glycerol kinase OS=Escherichia coli (strain K12)	93.32	54.78	1	25	25	32	3.567E8	502	56.2	5.50
P77804	Protein YdgA OS=Escherichia coli (strain K12)	91.30	58.17	1	23	23	29	9.258E7	502	54.7	5.17
P0CE47	Elongation factor Tu 1 OS=Escherichia coli (strain K12)	71.19	73.60	2	21	21	27	1.377E8	394	43.3	5.45
P0AFG6	Dihydrolipoyllysine-residue succinyltransferase component of 2-oxoglutarate dehydrogenase complex OS=E. coli (strain K12)	63.11	34.57	1	15	15	20	1.909E8	405	44.0	5.81

Supplementary Figure 1. Purification of f1pIV verified by **a)** SDS-PAGE and **b)** Western blot. Lane 1, Biorad Precision Plus All Blue Marker; lane 2, total soluble protein; lane 3, total membrane protein; lane 4, solubilized membrane protein; lanes 5-10, fractions from size exclusion chromatography. The f1pIV subunit is indicated with an orange arrowhead and the multimer by a blue arrowhead. Lane 1 (protein markers) was cut away and visualized with white light, hence the difference in colouring compared to the rest of the blot. **c)** Mass spectrometry analysis. The top ten proteins identified in the mass spectrometry analysis are shown, with f1pIV clearly being the most abundant protein present. Protein preparations, SDS-PAGE and Western blotting were repeated multiple times during sample optimisation (n>3). Mass spectrometry was performed once from the same sample that was used for cryoEM imaging.

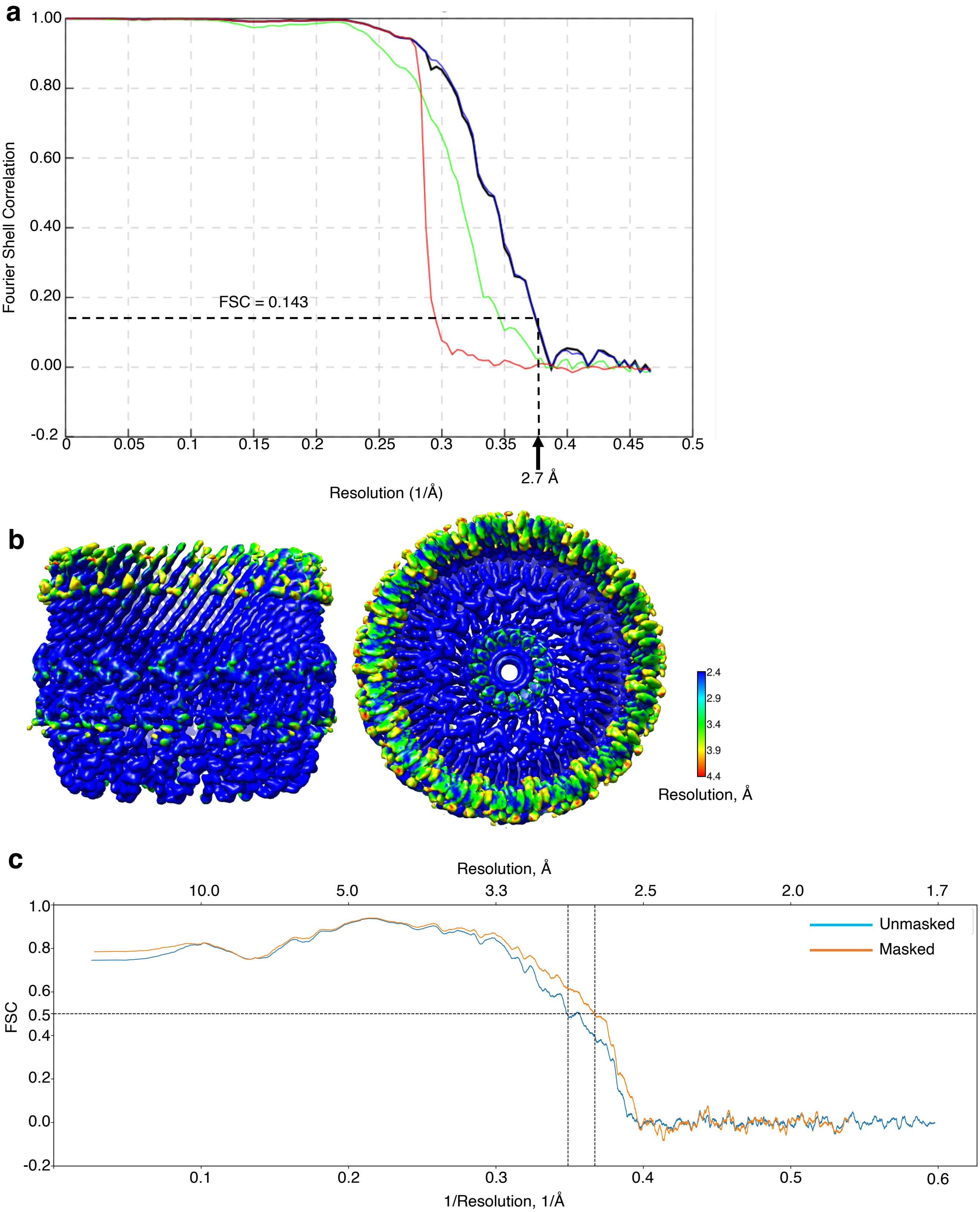

Supplementary Figure 2. f1pIV data quality. a) FSC curve obtained from Relion. The calculated final map was determined at 2.7 Å resolution using Fourier shell correlation at the 0.143 cut-off.
Red curve = rlnCorrectedFourierShellCorrelationPhaseRandomizedMaskedMaps.
Green curve = rlnFourierShellCorrelationUnmaskedMaps.
Blue curve = rlnFourierShellCorrelationMaskedMaps.
Black curve = rlnFourierShellCorrelationCorrected.
b) f1pIV cryoEM map coloured by local resolution (calculated with Resmap).
c) FSC curve showing the quality of the model to map fit (calculated with Phenix).

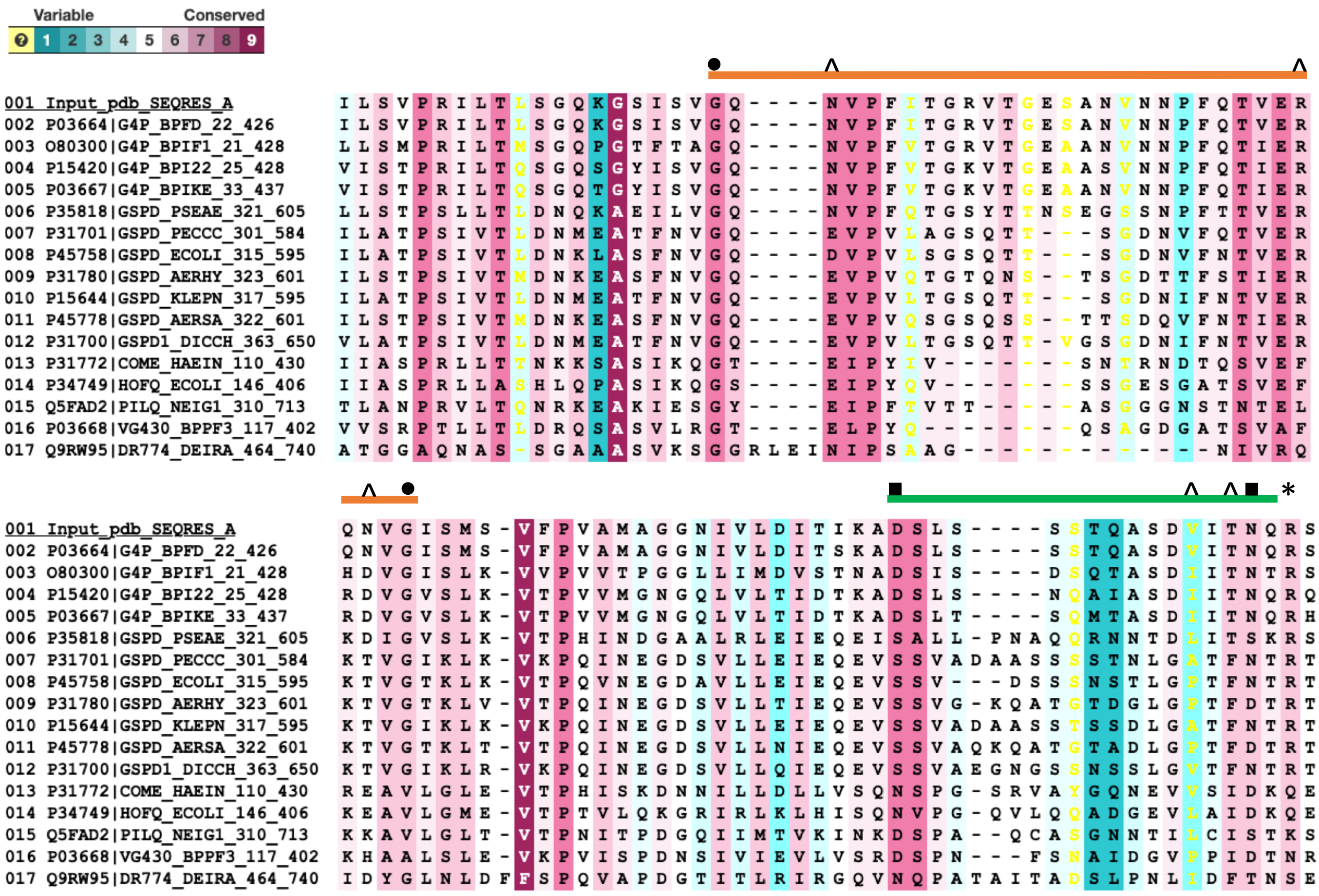

Supplementary Figure 3. Sequence alignment of the Gate region of 16 homologues from UniProtKB/Swiss-Prot coloured by conservation. Sequence 001 is f1pIV; 002-005 and 016 are other phage secretins. Gate 1 is indicated with an orange line and Gate 2 with a green line. Hinge residues Gly 267 and Gly 297 in the Gate 1 loop are denoted by circles and hinge residues Asp 321 and Asn 335 in the Gate 2 loop by squares. Residues involved in hydrogen bonding (Asn 269, Arg 293, Asn 295, Val 332, Thr 334; discussed in text and shown in Fig. 4b) are highlighted with arrow heads. A highly conserved Arg 337 found in a salt bridge in the interface between the inner and outer β-barrels is denoted by an asterisk.

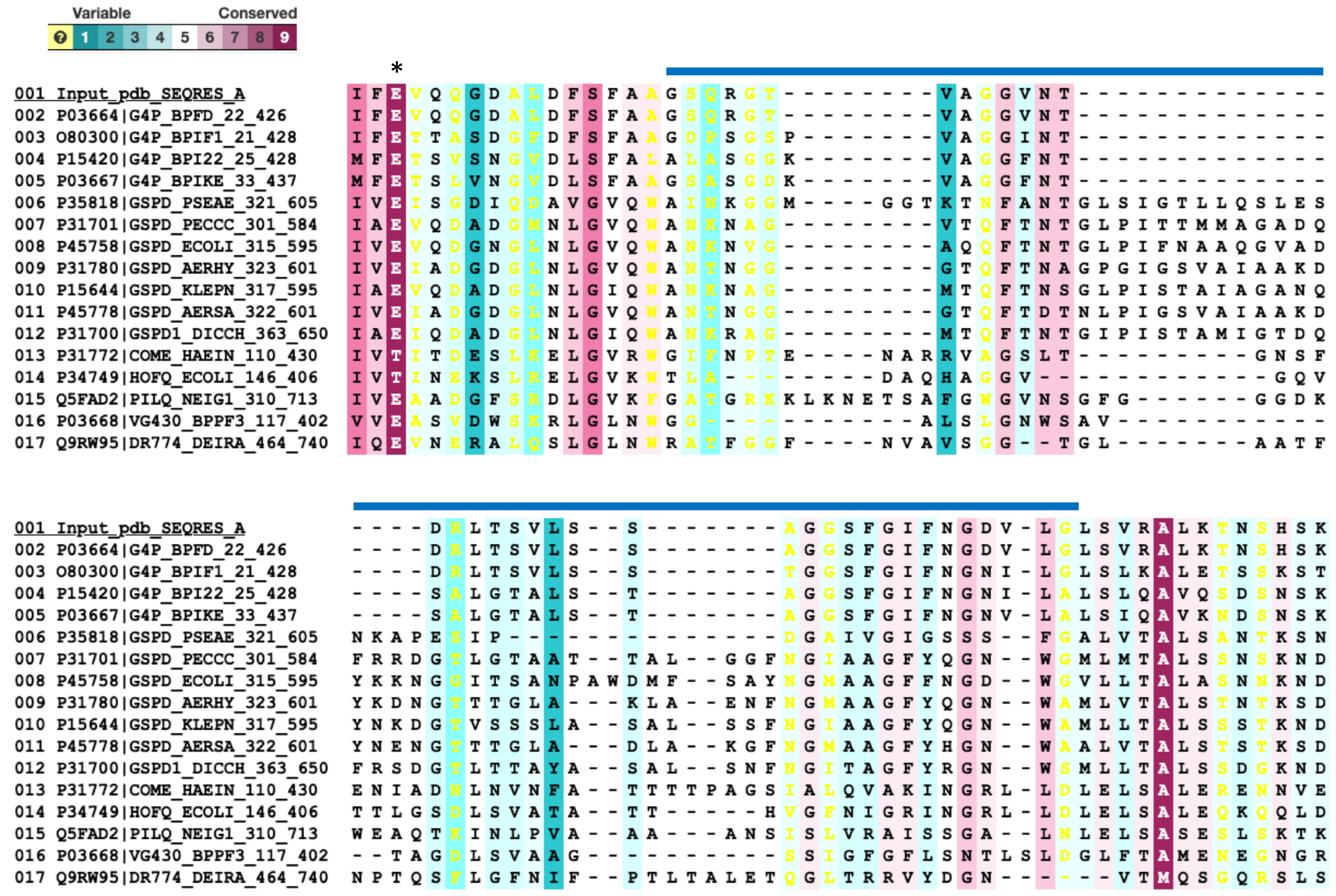

Supplementary Figure 4. Sequence alignment of the ß-barrel lip region of 16 homologues from UniProtKB/Swiss-Prot coloured by conservation. Sequence 001 is f1pIV; 002-005 and 016 are other phage secretins. The cap region is indicated with a blue line. Phage sequences have 36-37 residues in the ß-lip region. The remaining sequences are from bacterial Type II secretion systems and have 51-59 residues in this region. A highly conserved Glu 185 found at top of the interface between the inner and outer β-barrels is denoted by an asterisk.

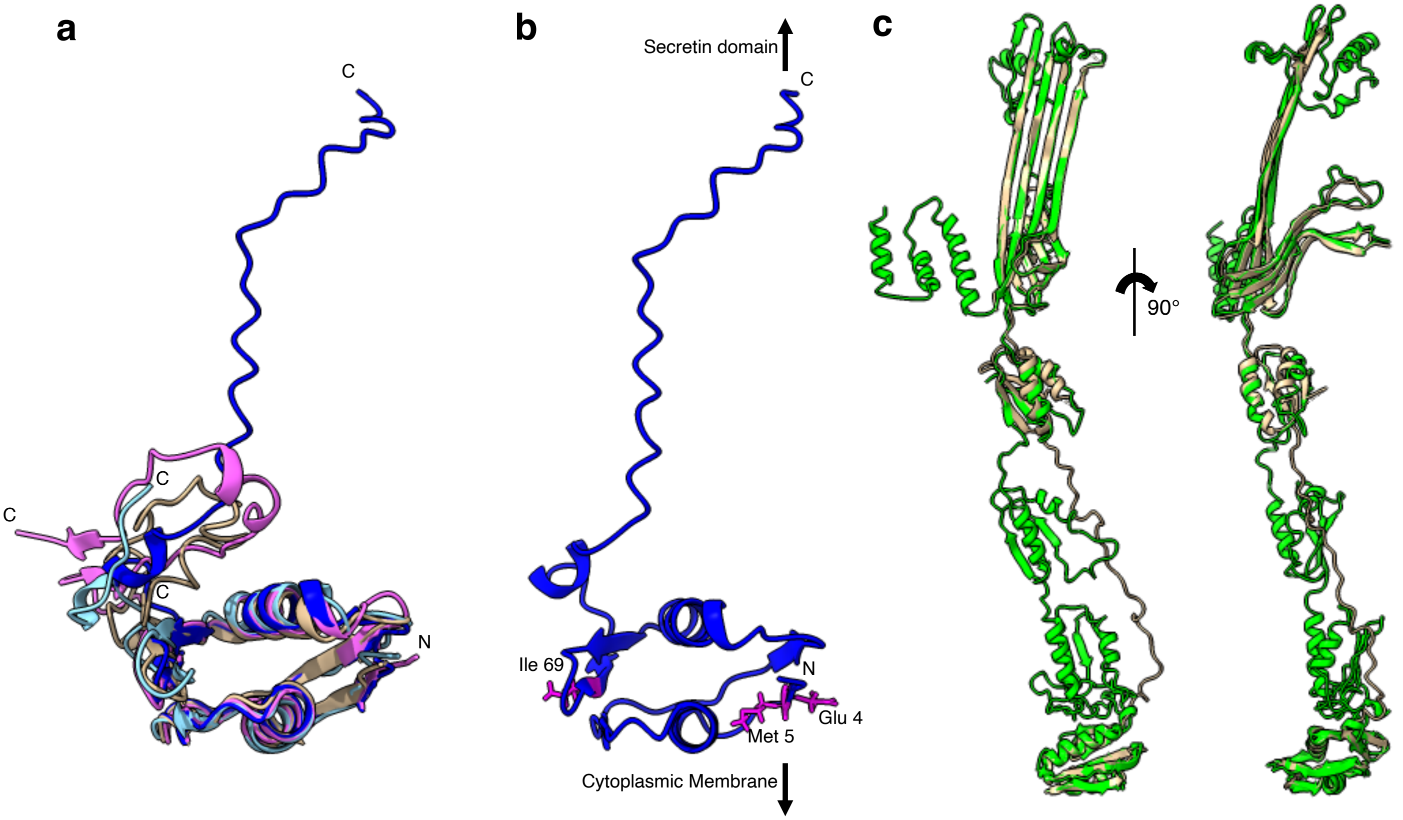

Supplementary Figure 5. Modelling the N0 domain. a) Structural superimposition of N0 homology models produced by Swiss-Model (light blue), I-TASSER (brown), Robetta (RoseTTAfold algorithm, magenta) and AlphaFold 2 (dark blue). The models are in agreement for residues 2-71 (the folded domain) and differ from residues 72-107 (the flexible linker). The amino termini are labelled N and the individual carboxyl termini are labelled C. **b)** The N0 domain model obtained from Alphafold with residues Glu 4, Met 5 and Ile 69 shown as magenta sticks and labelled. Arrows are included to show the relative positioning of the N0 domain within the periplasm. **c)** Structural superimposition of the PulD secretin from *Klebsiella pneumoniae* (green, 6HCG) with f1pIV composite model (brown).

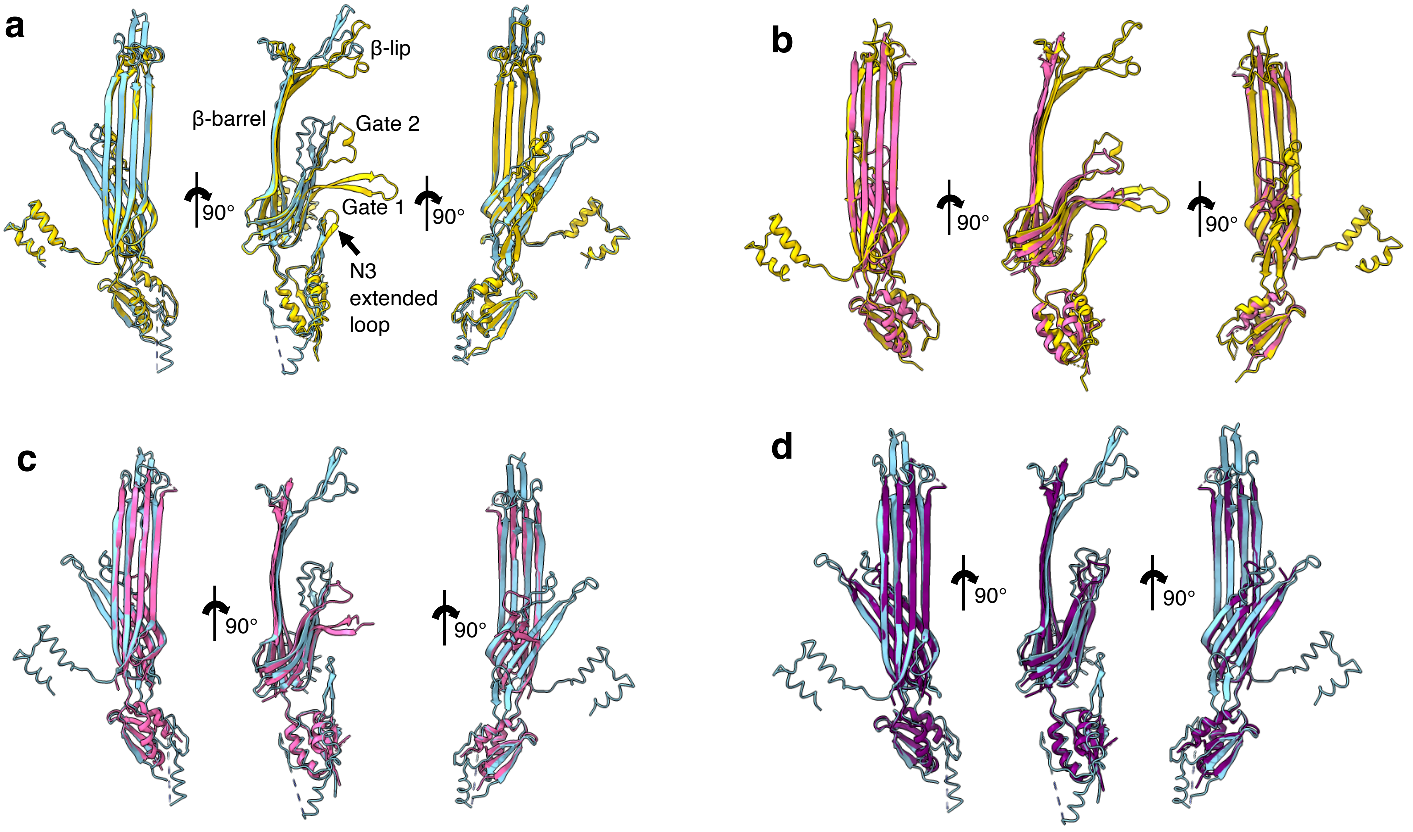

Supplementary Figure 6. Modelling f1pIV in the open state. a) Structural alignment of open (blue, 6Q15) and closed (yellow, 6PEE) states of InvG from *Salmonella typhimurium* in front, side and back views from left to right. **b)** Structural alignment of the closed state of InvG (yellow, 6PEE) and f1pIV (this study: pink, 7OFH) in front, side and back views from left to right. The average distance between the atoms of the two superimposed proteins was measured using the Root Mean Square Deviation (RMSD), giving a value of 1.051 Å between 94 pruned atom pairs. **c)** Structural alignment of the open state of InvG (blue, 6Q15) and f1pIV (this study: pink, 7OFH) in front, side and back views from left to right. **d)** Structural alignment of the open state of InvG (blue, 6Q15) and f1pIV modelled in an open form (purple) in front, side and back views from left to right.

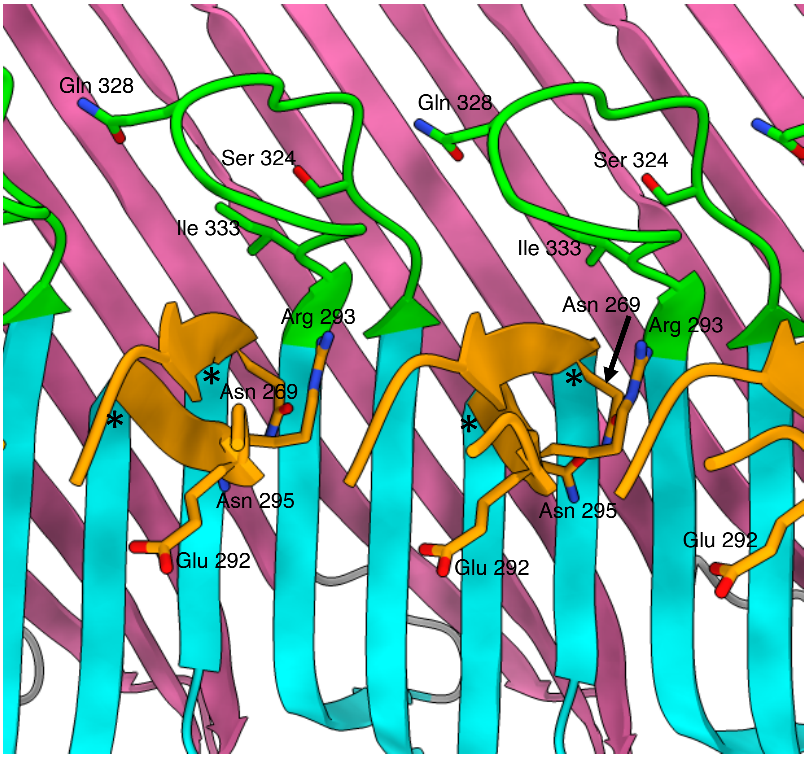

Supplementary Figure 7. f1pIV leaky mutants. An f1pIV 15-mer is shown as a cut-through, viewed from the centre of the pore. Residues Asn 269, Glu 292, Arg 293 and Asn 295 are shown in stick form in the Gate 1 loop (orange), residues Ser 324, Ile 333 and Gln 328 are shown in stick form in the Gate 2 loop (green). Hinge residues Gly 267 and Gly 297 are denoted by asterisks. Colouring as per Fig. 2b.

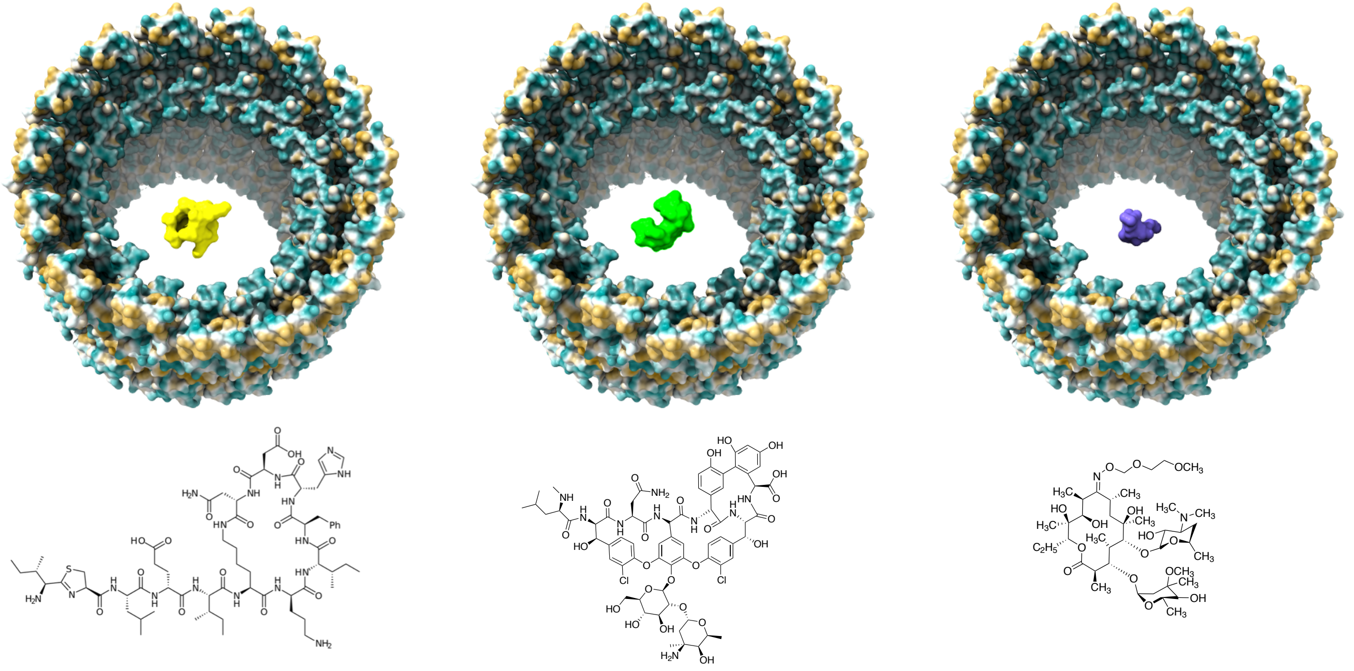

Bacitracin Vancomycin Roxithromycin

Supplementary Figure 8. Antibiotic uptake through open f1pIV. The open f1pIV model (coloured by hydrophobicity with the most hydrophobic areas coloured orange, through white, to the most hydrophilic in dark cyan) with bacitracin (yellow, amphipathic), vancomycin (lime green, hydrophilic) and roxithromycin (purple, hydrophobic) shown as surfaces. The chemical structures of each antibiotic are shown below.

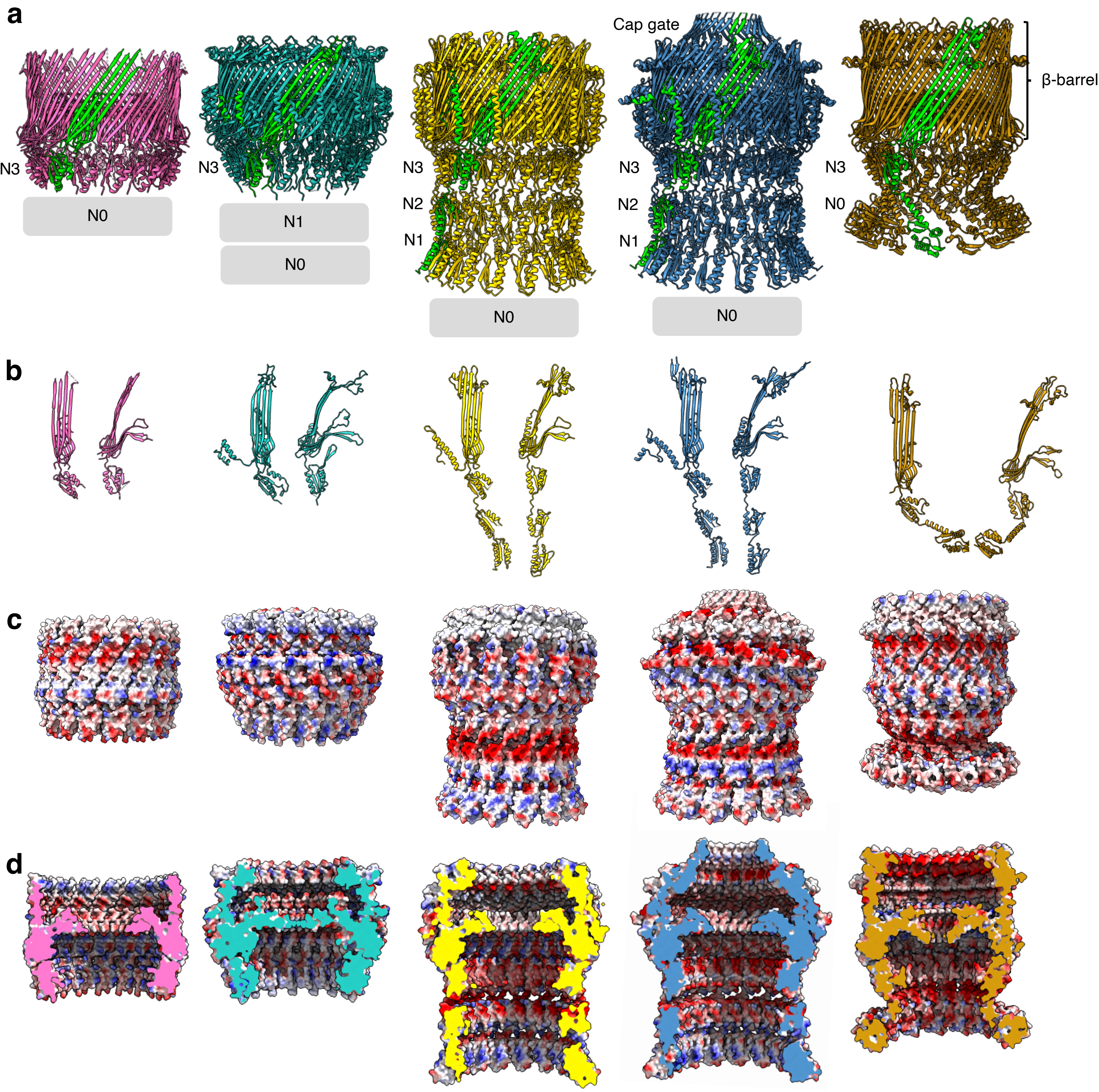

Supplementary Figure 9. Structural comparison of f1pIV with a selection of different classes of bacterial secretins. Secretins are aligned at the β-barrel domain. f1pIV is shown in pink (this study: 7OFH), the Type III secretin InvG from *Salmonella typhimurium* in sea green (6PEE), the Type II secretin GspD from *E. coli* K12 in yellow (*Klebsiella*-type, 5WQ7), the Type II secretin GspD from *Vibrio cholerae* in blue (*Vibrio*-type, 5WQ8) and the Type IV pilus secretin PilQ from *Vibrio cholerae* in orange (6W6M). The secretins are shown in **a)** as multimers (with one subunit coloured lime green). Periplasmic N domains not observed in the map are shown as grey boxes. The corresponding individual subunits are shown in **b)** in front and side view. **c)** Electrostatic surface potential comparison of multimers and in **d)** cut-through to show the inner surface inside the pore.

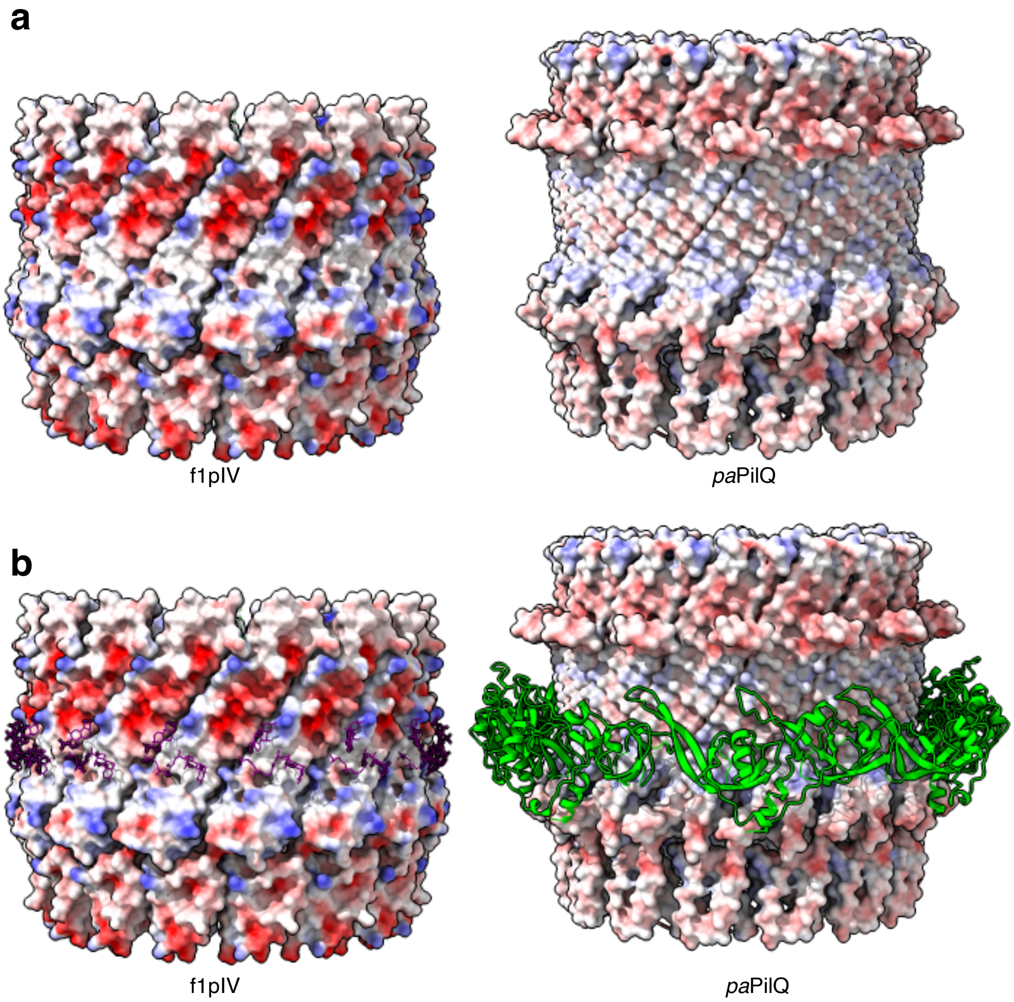

Supplementary Figure 10. Comparison of electrostatic surfaces and pilotin binding. Secretins are aligned at their β-barrel domains. **a)** Electrostatic surface charge representation of f1pIV (this study: 7OFH) and PilQ from *Pseudomonas aeruginosa* (*pa*PilQ) with pilotin removed (6VE2). **b)** Electrostatic surface charge representation of f1pIV showing CHAPS molecules bound (purple sticks) and *pa*PilQ showing the pilotin TsaP bound (cartoon representation in green).

Results colour-coded for amino acid conservation

The current colourscheme of the alignment is for **amino acid conservation**.

The conservation scoring is performed by PRALINE. The scoring scheme works from 0 for the least conserved alignment position, up to 10 for the most conserved alignment position.
The colour assignments are:

Unconserved 0 1 2 3 4 5 6 7 8 9 10 **Conserved**

```
            .........10 .........20 .........30 .........40 .........50
f1pIV       ---------- ---------QV IEMNNSPLRD FVTWYSKQTG ESVIVSPDVK
KpGspD      MKKLLASILL FFTFTVYAIP VELNNVPLRE FVSWYSKVSG QPVIVSPDVK
Consistency 0000000000 0000000012 8*7**2***6 **5****25* 63********

            .........60 .........70 .........80 .........90 .........100
f1pIV       GTVTVYSSDV KPE---NLRN FFISVLRANN FDMVGSNPSI IQKYNPNNQD
KpGspD      GEVTVYSADV KREELSQFFV S---VLRANG YDLSNGNPGV VQKY----SS
Consistency *3*****6** *2*0004411 2000*****4 6*7244**48 8***000044

            .........110.........120.........130.........140.........150
f1pIV       YIDELPSSDN QEYDDNSAPS GGF------- -FVPQNDNVT QTFKINNVRA
KpGspD      RNYEYADSFS DDVDYVPQDS SSAPAGDFFN PAKTQASLIT QTYPVNNVRA
Consistency 210*334*15 463*01332* 4420000000 0223*2418* **638*****

            .........160.........170.........180.........190.........200
f1pIV       KDLIRVVELF VKSNTSKSSN VLSIDGSNLL VVSAPKDILD NLPQFLSTVD
KpGspD      KDLAPVIDVF LKGDNIAGTK VFPFDGANIL AVTASASQHK QLKEFFPSVD
Consistency ***32*866* 6*45423454 *434**6*7* 5*5*334113 4*36*435**

            .........210.........220.........230.........240.........250
f1pIV       LPTDQILIEG LIFEVQQGDA LDFSFAAGSQ RGT-VAGGVN TDRLTSVLSS
KpGspD      VPRIQVLVES VIFETSASDG FDFSFAAGDP SGHPIAGGVN TDRLGKVLSS
Consistency 6*31*8*8*4 6***4434*4 4*******43 3*108***** ****24****

            .........260.........270.........280.........290.........300
f1pIV       AGGSFGIFNG DVLGLSVRAL KTNSHSKILS VPRILTLSGQ KGSISVGQNV
KpGspD      SGGSFGIFNG NVLALSLKAL ETSNKSTLLS MPRILTMSGQ PGTFTAGQNV
Consistency 6********* 5**4**66** 5*552*37** 5*****7*** 3*5455****

            .........310.........320.........330.........340.........350
f1pIV       PFITGRVTGE SANVNNPFQT IERQNVGISM SVFPVAMAGG NIVLDITSKA
KpGspD      PFVTGRITGE AASVNNPFQT IERRDVGISL QVVPVVTPAG LLIMDVKTNA
Consistency **8***8*** 6*5******* ***55****7 4*3**5334* 1787*8354*

            .........360.........370.........380.........390.........400
f1pIV       DSLSSSTQAS DVITNQRSIA TTVNLRDGQT LLLGGLTDYK NTSQDSGVPF
KpGspD      DSISDSQAAS DIITNTRSIS TTVQLKSGQT VLLGGMVDNR DSEADSSVPW
Consistency **7*4*33** *8***3***6 ***4*64*** 6****74*16 5543**4**4

            .........410.........420.........430.....
f1pIV       LSKIPLIGLL FSSRSDSNEE STLYVLVKAT IVRAL
KpGspD      VSKIPLIGAL FTSKSTNASK RTLYMLIRAR VVNPL
Consistency 6*******3* *5*6*35245 3***5*86*3 8*43*
```

Supplementary Figure 11. Sequence alignment performed with Praline. The closest homologue of f1pIV based on sequence identity is GspD from *Klebsiella pneumoniae.*

Data collection and processing	Dataset 1	Dataset 2
Acceleration voltage (kV)	300	300
Nominal magnification (x)	81k	81k
Pixel size (Å)	1.072	0.536 (super-res)
Frame rate (s^{-1})	11.93	11.93
Exposure time (s)	3.52	3.52
Total exposure (e^-/Å)	42.059	42.059
Particles		
Micrographs used for selection	7,037	14,336
Defocus range (µm)	-2.5 to -1.3 (0.2 µm steps)	-2.5 to -1.3 (0.2 µm steps)
Total number of particles	241,591	330,389
In final 3D reconstruction	16,104	95,575
	Dataset 1 and Dataset 2 combined	
Resolution		
"Gold-standard" at FSC 0.143 (Å)	2.7	
Map-sharpening B factor ($Å^2$)	0	
Model refinement		
Model composition	15 identical subunits	
Non-hydrogen atoms, per chain	1,742	
Protein residues, per chain	231	
Ligands, per chain	2	
R.M.S. Z scores		
Bond lengths (Å)	0.48	
Bond angles (°)	0.72	
R.M.S.D		
Bond lengths (Å)	0.031	
Bond angles (º)	1.903	
Validation		
Ramachandran angles (%)		
Favoured	88	
Allowed	12	
Outliers	0	
Clashscore	14	
Rotamer outliers (%)	2	
Resolution estimates (Å)		
Model resolution (0/0.143/0.5) - masked	2.5/2.6/2.7	
Model resolution (0/0.143/0.5) - unmasked	2.4/2.5/2.9	

Supplementary Table 1. The statistics of data collection, model reconstruction, refinement and validation.

f1pIV leaky mutation	Location in structure	Leaky to maltopentaose sugars /deoxycholate	Leaky (sensitive) to vancomycin	Leaky (sensitive) to bacitracin
**A121V**	N3 domain	✓	×	×
**D123Y**	N3 domain	✓	×	×
**G147V**	N3 domain	✓	×	×
**I183V**	Secretin domain	✓	×	×
G259S/D	Gate region	✓	×	×
S263F	Gate region	✓	✓	✓
**G267D**	Gate region	✓	×	×
**N269D**	Gate region	✓	✓	✓
V270I	Gate region	✓	✓	✓
P271S	Gate region	✓	×	×
I273V	Gate region	✓	×	×
G275D	Gate region	✓	×	×
R276C	Gate region	✓	×	×
V277A	Gate region	✓	×	×
G279D	Gate region	✓	×	×
S281P	Gate region	✓	×	×
A282G	Gate region	✓	✓	×
N283K	Gate region	✓	×	×
V284A	Gate region	✓	×	×
F288L	Gate region	✓	×	×
**E292K**	Gate region	✓	✓	✓
**R293C**	Gate region	✓	×	×
**N295S**	Gate region	✓	✓	✓
V296I	Gate region	✓	×	×
**G297V**	Gate region	✓	×	×
S322N	Gate region	✓	✓	✓
**S324G**	Gate region	✓	✓	✓
S325P	Gate region	✓	✓	✓
S326F	Gate region	✓	✓	×
T327A	Gate region	✓	✓	×
**Q328G**	Gate region	✓	✓	✓
A329T/V	Gate region	✓	×	×
S330N	Gate region	✓	×	×
D331N	Gate region	✓	✓	✓
**I333V**	Gate region	✓	✓	×
T334A	Gate region	✓	✓	✓

Supplementary Table 2. Leaky f1pIV mutants. The leaky mutants from Spagnuolo *et al*[20] were plotted onto the f1pIV structure shown in Fig. 5a and b. Residues discussed at various points in the main text are shown in bold.
