# Supplementary material for: CryoEM structure of the outer membrane secretin channel pIV from the f1 filamentous bacteriophage
Source: Nat Commun. 2021 Nov 2;12:6316. doi: 10.1038/s41467-021-26610-3 (PMC8563730; doi:10.1038/s41467-021-26610-3)
Supplement: Supplementary file 3 — Reporting Summary [file 41467_2021_26610_MOESM3_ESM.pdf]

nature portfolio

Corresponding author(s): Vicki Gold

Last updated by author(s): Oct 5, 2021

Reporting Summary

Nature Portfolio wishes to improve the reproducibility of the work that we publish. This form provides structure for consistency and transparency in reporting. For further information on Nature Portfolio policies, see our Editorial Policies and the Editorial Policy Checklist.

Statistics

For all statistical analyses, confirm that the following items are present in the figure legend, table legend, main text, or Methods section.

n/a	Confirmed	
☐	☒	The exact sample size (*n*) for each experimental group/condition, given as a discrete number and unit of measurement
☐	☒	A statement on whether measurements were taken from distinct samples or whether the same sample was measured repeatedly
☐	☒	The statistical test(s) used AND whether they are one- or two-sided<br>*Only common tests should be described solely by name; describe more complex techniques in the Methods section.*
☒	☐	A description of all covariates tested
☒	☐	A description of any assumptions or corrections, such as tests of normality and adjustment for multiple comparisons
☒	☐	A full description of the statistical parameters including central tendency (e.g. means) or other basic estimates (e.g. regression coefficient) AND variation (e.g. standard deviation) or associated estimates of uncertainty (e.g. confidence intervals)
☐	☒	For null hypothesis testing, the test statistic (e.g. *F*, *t*, *r*) with confidence intervals, effect sizes, degrees of freedom and *P* value noted<br>*Give P values as exact values whenever suitable.*
☒	☐	For Bayesian analysis, information on the choice of priors and Markov chain Monte Carlo settings
☒	☐	For hierarchical and complex designs, identification of the appropriate level for tests and full reporting of outcomes
☒	☐	Estimates of effect sizes (e.g. Cohen's *d*, Pearson's *r*), indicating how they were calculated

Our web collection on statistics for biologists contains articles on many of the points above.

Software and code

Policy information about availability of computer code

Data collection	EPU 2.81 (ThermoFisher Scientific), Xcalibur 2.1 (Thermo Scientific)
Data analysis	Warp 1.0.9, Relion 3.1, Chimera 1.14, Coot 0.9.3, CCPEM 1.5.0, DeepEMhancer (Sep 2020), Phenix vdev-3699, ProteomeDiscoverer 1.4 (Thermo Scientific), IMOD 4.11.6

For manuscripts utilizing custom algorithms or software that are central to the research but not yet described in published literature, software must be made available to editors and reviewers. We strongly encourage code deposition in a community repository (e.g. GitHub). See the Nature Portfolio guidelines for submitting code & software for further information.

Data

Policy information about availability of data

All manuscripts must include a data availability statement. This statement should provide the following information, where applicable:

- Accession codes, unique identifiers, or web links for publicly available datasets
- A description of any restrictions on data availability
- For clinical datasets or third party data, please ensure that the statement adheres to our policy

Density maps of f1pIV have been deposited in the Electron Microscopy Data Bank (EMDB), with accession number EMD-12874. An atomic model of f1pIV has been deposited in the Protein Data Bank (PDB) with accession number 7OFH.
The source image data used in this study have been deposited in the Electron Microscopy Public Image Archive (EMPIAR) under accession number EMPIAR-10807.
UniProt was also used in this study for mass spectrometry analysis (Escherichia coli database (4,349 sequences)).

Field-specific reporting

Please select the one below that is the best fit for your research. If you are not sure, read the appropriate sections before making your selection.

☒ Life sciences ☐ Behavioural & social sciences ☐ Ecological, evolutionary & environmental sciences

For a reference copy of the document with all sections, see nature.com/documents/nr-reporting-summary-flat.pdf

Life sciences study design

All studies must disclose on these points even when the disclosure is negative.

Sample size	No specific statistical methods were used to determine sample size as it was not generally applicable to our study. For electron microscopy data, sample size was determined by the availability of areas to image on grids. For microfluidics experiments, sample size was determined by the number of cells available to image within the timeframe of the experiment. In both cases, sample sizes are sufficiently large to be statistically relevant (n = 21,373 images and 571,980 particles; n = 501 cells)
Data exclusions	No data have been excluded
Replication	n = 501 single cells; 241 for WT pIV and 260 for pIV S324G in the data shown in Fig. 5d.
Randomization	Randomization was not relevant as particles used for structural determination were selected automatically, and all cells for microfluidic analysis were included in the analysis.
Blinding	Blinding was not relevant since no data was excluded and possible biased evaluation steps were calculated using established software.

Reporting for specific materials, systems and methods

We require information from authors about some types of materials, experimental systems and methods used in many studies. Here, indicate whether each material, system or method listed is relevant to your study. If you are not sure if a list item applies to your research, read the appropriate section before selecting a response.

Materials & experimental systems

n/a	Involved in the study	
☐	☒	Antibodies
☒	☐	Eukaryotic cell lines
☒	☐	Palaeontology and archaeology
☒	☐	Animals and other organisms
☒	☐	Human research participants
☒	☐	Clinical data
☒	☐	Dual use research of concern

Methods

n/a	Involved in the study	
☒	☐	ChIP-seq
☒	☐	Flow cytometry
☒	☐	MRI-based neuroimaging

Antibodies

Antibodies used	Primary antibody: rabbit anti-f1pIV. This antibody was produced in-house at Rockefeller University and was a gift from Marjorie Russel.<br>Secondary antibody: Biorad blotting grade goat anti-rabbit IgG horseradish peroxidase conjugate (catalogue number 1706515)
Validation	Primary antibody: anti f1pIV antibody. This is described in Brisette & Russel (1990), Journal of Molecular Biology, Volume 211, Issue 3, Pages 565-580; Spagnuolo et al (2010), Molecular Microbiology, Volume 76, Issue 1, Pages 133-150<br><br>Secondary antibody: anti-rabbit IgG horseradish peroxidase.<br>https://www.bio-rad.com/en-uk/sku/1706515-goat-anti-rabbit-igg-h-l-hrp-conjugate?ID=1706515
